# Investigation into CYP3A4-mediated drug–drug interactions on midostaurin in healthy volunteers

**DOI:** 10.1007/s00280-013-2287-6

**Published:** 2013-10-02

**Authors:** Catherine Dutreix, Florence Munarini, Sebastien Lorenzo, Johannes Roesel, Yanfeng Wang

**Affiliations:** 1 Novartis Oncology, Basel, Switzerland; 2 Novartis Oncology, East Hanover, NJ USA; 3 Novartis Pharma AG, Postfach, CH-4002 Basel, Switzerland

**Keywords:** FMS-related tyrosine kinase 3 (FLT3) inhibitor, Ketoconazole, Midazolam, Midostaurin, Pharmacokinetics, Rifampicin

## Abstract

**Purpose:**

Midostaurin (PKC412), a multitargeted tyrosine kinase inhibitor that targets FMS-related tyrosine kinase 3 and KIT, is in clinical trials for the treatment for acute myeloid leukemia and advanced systemic mastocytosis. In vitro studies showed that midostaurin is predominantly metabolized by cytochrome P450 3A4 (CYP3A4) and that midostaurin inhibits and/or induces the same enzyme. Here, we address the clinical relevance of CYP3A4-related drug–drug interactions with midostaurin as either a “victim” or “perpetrator.”

**Methods:**

Three phase I studies in healthy volunteers evaluated the effects of a CYP3A4 inhibitor (ketoconazole 400 mg daily for 10 days) or CYP3A4 inducer (rifampicin 600 mg daily for 14 days) on concentrations of midostaurin and its metabolites following a single 50-mg dose of midostaurin and the effects of midostaurin as a single dose (100 mg) and multiple doses (50 mg twice daily) on midazolam (a sensitive CYP3A4 probe) concentration. The plasma concentrations of midostaurin and its 2 active metabolites, CGP62221 and CGP52421, were determined using a sensitive liquid chromatography/tandem mass spectrometry method.

**Results:**

Inhibition of CYP3A4 by ketoconazole increased midostaurin exposure more than tenfold, and induction of CYP3A4 by rifampicin decreased midostaurin exposure by more than tenfold. Midostaurin did not appreciably affect the concentrations of midazolam or its metabolite, 1′-hydroxymidazolam, at single or multiple doses.

**Conclusion:**

The pharmacokinetics of midostaurin and its metabolites was affected substantially by ketoconazole and rifampicin, suggesting that midostaurin is a sensitive CYP3A4 substrate. Midostaurin did not appear to inhibit or induce CYP3A4 in vivo.

**Electronic supplementary material:**

The online version of this article (doi:10.1007/s00280-013-2287-6) contains supplementary material, which is available to authorized users.

## Introduction

Midostaurin, an orally bioavailable staurosporine analogue, is a multitargeted tyrosine kinase inhibitor that targets FMS-related tyrosine kinase 3 (FLT3), wild-type and mutant KIT, platelet-derived growth factor receptor β, and other receptors [1–3]. Midostaurin is metabolized in humans primarily via cytochrome P450 3A4 (CYP3A4) into two major pharmacologically active circulating metabolites, CGP62221 (via *O*-demethylation) and CGP52421 (via 7-hydroxylation) [4–6]. In vitro studies have shown that midostaurin, CGP62221, and CGP52421 inhibit mutant FLT3 at low nanomolar concentrations, with half-maximal inhibitory concentrations (IC_50_) of 10–36, 26, and 584 nM, respectively [3, 4]. The apparent elimination half-life (*t*_1/2_) of CGP52421 is long, ranging from 16 to 31 days [5, 6]; thus, CGP52421 may contribute to sustained in vivo activity.

Mutations in *FLT3* and *KIT* have been shown to play a role in the development of malignancies such as acute myeloid leukemia (AML) and advanced systemic mastocytosis (ASM) [7–9]. Approximately 30 % of patients with AML have *FLT3* mutations that lead to constitutive activation [1]. Midostaurin has demonstrated clinical activity as a single agent or in combination with chemotherapy in patients with AML [10, 11] and is currently under investigation in a phase III clinical trial in patients with newly diagnosed AML who have a mutation in *FLT3* [12]. Most patients with ASM (≈80 %) have gain-of-function mutations in *KIT*, particularly the D816V mutation [13]. A phase II investigator-initiated study of midostaurin 100 mg twice daily in 26 patients with ASM demonstrated a response rate of 69 % according to Valent criteria [14], including 10 major responses (6 incomplete remissions and 4 pure clinical responses); 5 good partial responses; and 3 minor partial responses [15]. Strong responses were observed in patients with the *KIT* D816V mutation, the majority of whom achieved a major response [15]. Furthermore, a 60 % overall response rate (53 % major response) was reported in the stage 1 analysis of an ongoing global phase II study of midostaurin in patients with ASM or mast cell leukemia with or without an associated clonal hematologic non-mast cell lineage disease, the majority of whom (70 %) were *KIT* D816V/Y-positive [16].

Previous studies have evaluated the pharmacokinetics (PK) of midostaurin in patients with AML [11, 17] and diabetes [6]. These studies showed that midostaurin is rapidly absorbed after oral administration, with peak plasma concentrations observed at 1–3 h postdose. A separate study in healthy volunteers found that the mean apparent volume of distribution of midostaurin (111 L) was higher than that of total body water (42 L), indicating a high tissue distribution [18]. The maximal concentration (*C*_max_) and area under the concentration curve (AUC) increased proportionally with dose following single-dose administration of midostaurin between 25- and 50-mg doses but under proportionally between 50- and 100-mg doses [6].

Upon daily oral dosing, midostaurin concentrations accumulated in a time-linear manner in the first 3–6 days. Thereafter, the PK becomes time-dependent; the plasma concentrations of midostaurin decreased substantially between days 6 and 14 and reached a new plateau after 2–3 weeks of daily dosing [18]. This concentration drop over time necessitates twice-daily administration of midostaurin to maintain long-term target drug levels. The concentrations of CGP62221 metabolite showed a similar time profile as the parent drug midostaurin. In contrast, plasma levels of the metabolite CGP52421 rose over treatment cycles, as expected by its long half-life of >1 month [5, 6].

The long plasma half-lives of midostaurin and its metabolites could be due to their specific and strong binding to the human serum protein α1-acid glycoprotein (AAG). Midostaurin, CGP62221, and CGP52421 are 88–98 % bound to AAG [19], resulting in free midostaurin concentrations of approximately 0.02–1 μM [18]. Fecal excretion is the major pathway for the elimination of midostaurin, CGP62221, and CGP52421 [18].

Given the prominent role of CYP3A4 in midostaurin metabolism, drug–drug interactions (DDIs) that affect the exposure of midostaurin and its metabolites may occur when midostaurin is co-administered with potent CYP3A4 inhibitors or inducers. Furthermore, midostaurin and its major metabolites inhibit CYP3A4/5 activity in vitro (midazolam 1′-hydroxylation, IC_50_ = 1.5 μM for midostaurin and CGP52421 and IC_50_ <1 μM for CGP62221) [18] and activate the pregnane X receptor at concentrations of 1–50 μmol/L in a CYP3A4 reporter gene assay [6]. Therefore, it is possible that midostaurin and/or its metabolites may affect the metabolic clearance of comedications metabolized by CYP3A4.

To address the clinical relevance of CYP3A4-related DDIs at clinically relevant doses, a series of studies was performed in healthy volunteers. Herein, we describe the results of these studies that evaluated the effects of a CYP3A4 inhibitor (ketoconazole) or CYP3A4 inducer (rifampicin) on concentrations of midostaurin and its metabolites and the effects of midostaurin on the PK of midazolam (a sensitive CYP3A4 probe).

## Methods

### Participants

All studies enrolled healthy volunteers. Eligible individuals were aged 18–55 years, weighed 50–90 kg, had a body mass index of 18–29.9 kg/m^2^ (upper limit of 30 kg/m^2^ for the ketoconazole study), and had normal blood pressure. Volunteers were excluded from participation if they tested positive for HIV, hepatitis B, or hepatitis C or if they had an abnormal electrocardiogram, a history of myocardial infarction, or a family medical history of long QT interval syndrome. Women currently pregnant or breast-feeding were excluded, as were individuals who had used any prescription medication within 14 days or over-the-counter medication in the past 7 days. Additionally, participants had to be willing to abstain from sexual intercourse or use an approved form of contraception while on study. Additional inclusion/exclusion criteria are included in Supplementary Table 1.

### Study designs and objectives

#### Midostaurin PK when administered with ketoconazole

In this single-center, open-label, randomized, parallel-group phase I study, healthy volunteers were randomized to receive either ketoconazole 400 mg or placebo daily from day 1 to day 10; in addition, all participants received midostaurin 50 mg once on day 6 (Table 1). According to the US Food and Drug Administration, ketoconazole 400 mg once daily is the recommended dose to evaluate DDIs involving CYP3A4 inhibition [20].
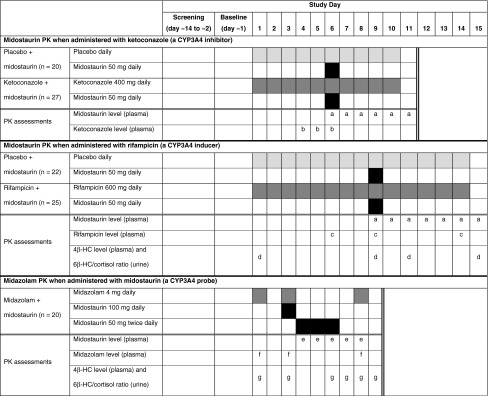

**Table 1:**
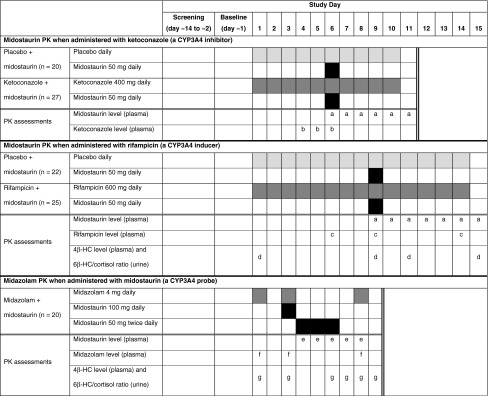
Study designs

Black highlight indicates midostaurin. Medium gray highlight indicates CYP3A4 inhibitor, inducer, or probe. Light gray highlight indicates placebo. Double vertical lines indicate the end of a given study

*4β*-*HC* 4β-hydroxycholesterol, *6β*-*HC* 6β-hydroxycortisol, *CYP3A4* cytochrome P450 3A4, *PK* pharmacokinetics

^a^Blood samples for the determination of midostaurin PK were collected predose and at 0.5, 1, 1.5, 2, 3, 4, 6, 8, 12, 24, 36, 48, 72, 96, and 120 h after midostaurin dosing on day 6 (ketoconazole study) and day 9 (rifampicin study). In the rifampicin study, an additional sample was taken at 144 h after midostaurin dosing on day 9

^b^On days 4–6, blood samples were collected before the morning dose to measure ketoconazole levels

^c^On days 6, 9, and 14, blood samples were collected for rifampicin assessment before the evening rifampicin dose

^d^On days 1 (baseline), 9 (before midostaurin treatment), 11, and 15, levels of 4β-HC in plasma and the ratio of 6β-HC/cortisol in urine were assessed

^e^Blood samples for PK determination of midostaurin and its metabolites (CGP62221 and CGP52421) were taken before the morning dose of midostaurin on days 4–6 and before midazolam administration on days 7 and 8

^f^Blood samples for the determination of midazolam PK were collected before dose and at 0.25, 0.5, 1, 1.5, 1.75, 2, 3, 4, 6, 8, and 10 h after midazolam dosing on days 1, 3, and 8

^g^Blood samples and urine samples were taken on days 1, 3, 6, 7, 8, and 9 to measure 4β-HC in plasma and the ratio of 6β-HC/cortisol in urine, respectively

The primary objective of this study was to investigate the effect of ketoconazole on the PK of a single oral dose of midostaurin in healthy volunteers. The secondary objective was to assess the safety and tolerability of a single dose of midostaurin given alone or in combination with ketoconazole in healthy volunteers. All randomized participants were included in the population evaluated for safety, whereas the PK population only included individuals who received all planned doses of the study drug, had evaluable PK profiles, and did not vomit within 4 h after midostaurin administration. Adverse events (AEs) were recorded during the study and up to 1 month (28 days) after the end of study. Volunteers remained at the clinic until AEs were common toxicity criteria (CTC) grade ≤1. Blood samples (4 mL/sample) for the determination of midostaurin PK and ketoconazole levels were collected predose and at various times after midostaurin dosing on day 6 (Table 1).

#### Midostaurin PK when administered with rifampicin

In this open-label, randomized, parallel-group phase I study, healthy volunteers were randomized to receive either rifampicin 600 mg or placebo daily from day 1 to day 14; all participants received midostaurin 50 mg once on day 9 (Table 1). The primary objective of this study was to investigate the effect of co-administration of rifampicin on the PK of a single oral dose of midostaurin in healthy volunteers. The secondary objective was to investigate the safety and tolerability of midostaurin alone and in combination with rifampicin. The PK and safety populations and safety assessments used the same definitions as those in the ketoconazole study.

Blood samples were collected at various times to assess midostaurin PK and rifampicin levels (Table 1). In addition, on days 1 (baseline), 9 (before midostaurin treatment), 11, and 15, levels of 4β-hydroxycholesterol in plasma and the 6β-hydroxycortisol/cortisol ratio in urine were assessed. These endogenous molecules served as exploratory biomarkers for CYP3A4 activity in vivo [21, 22].

#### Midazolam PK when administered with midostaurin

In this open-label, single-arm phase I study, healthy volunteers were administered the following treatment regimen: on day 1, participants received a solution of midazolam 4 mg with a standard breakfast; day 2 was a rest day during which no treatment was administered; on day 3, participants received midostaurin 100 mg plus a solution of midazolam 4 mg with a standard breakfast (Table 1). On days 4–6, participants received midostaurin 50 mg twice daily with a standard breakfast; day 7 was a rest day with no treatment administered; on day 8, participants received a solution of midazolam 4 mg with a standard breakfast. The drug holiday on day 7 was added to minimize the inhibitory effect of midostaurin on CYP3A4 prior to midazolam dosing on day 8. Such a holiday is feasible without affecting enzyme induction because of the long half-lives of midostaurin and its metabolites [23] and the long degradation half-life of CYP3A4 (approximately 3 days) [24].

The primary objective of this study was to investigate the potential effect of midostaurin as “perpetrator” on the PK of midazolam as an inhibitor on day 3 and a potential inducer on day 8. Both inhibition and induction of midazolam clearance were investigated. The secondary objective was to investigate the safety and tolerability of concomitant administration of midazolam and midostaurin. All volunteers were included in the population evaluated for safety, whereas the PK population only included individuals who took all scheduled doses of midostaurin and midazolam without vomiting within 4 h after dosing.

Blood samples were collected at various time points to assess midazolam PK as well as levels of midostaurin and its metabolites (Table 1). Similarly, blood and urine samples were taken on days 1, 3, 6, 7, 8, and 9 to measure the levels of 4β-hydroxycholesterol in plasma and the 6β-hydroxycortisol/cortisol ratio in urine, markers of CYP3A4 activity.

### PK and statistical data analyses

#### DDI with ketoconazole

The PK parameters of midostaurin and its metabolites [*C*_max_, time to reach maximal concentration (*t*_max_), AUC_0–last_, AUC_inf_, and *t*_1/2_] were derived using noncompartmental methods with the aid of WinNonLin 5.2 software (Pharsight Corp, St Louis, MO, USA). Because of the long apparent elimination *t*_1/2_ of CGP52421, it was not possible with the given sampling schedule to reliably estimate AUC_0–inf_ and *t*_1/2_ values for this analyte. Following log transformation, AUC_0–last_ and *C*_max_ were analyzed separately using an analysis of variance (ANOVA) model including a term for treatment (“ketoconazole + midostaurin” as test and “placebo + midostaurin” as reference). A point estimate and the corresponding 90 % CI for the treatment effect were calculated for the test treatment compared with the reference. These were anti-logged to obtain the point estimate and the 90 % CI for the geometric mean ratio (GMR) on the untransformed scale. AUC_0–inf_ was also analyzed for midostaurin and its active metabolite CGP62221 using the same method. All ANOVAs were performed using SAS software (SAS Institute Inc., Cary, NC, USA).

#### DDI with rifampicin

The PK parameters of midostaurin and its metabolites (*C*_max_, *t*_max_, AUC_0–last_, AUC_inf_, *t*_1/2_, and Cl/F) were derived using noncompartmental methods with the aid of WinNonLin 5.2. As in the ketoconazole study, the sampling schedule was not consistent with obtaining good estimates of the apparent terminal *t*_1/2_ and consequently AUC_inf_ for CGP52421. Following log transformation, the primary variables were analyzed separately using an ANOVA model including a term for treatment (“rifampicin + midostaurin” as test and “midostaurin + placebo” as reference). A point estimate and the corresponding 90 % CI for the treatment effect (test vs reference) were calculated. These were anti-logged to obtain the GMR and respective 90 % CI. The *t*_max_ for midostaurin (and its metabolites) was compared between the test and reference treatments by comparing the median, minimum, and maximum values in the two treatment conditions. The median difference was estimated with the Hodges–Lehmann estimator and its respective exact two-sided 90 % CI. All formal comparative statistical analyses were performed using SAS software.

#### DDI with midazolam

The PK parameters of midazolam and its metabolites (*C*_max_, *t*_max_, AUC_0–last_, AUC_inf_, and *t*_1/2_) were derived using a noncompartmental method with the aid of WinNonLin 5.2. In this study, the reference treatment was midazolam administered alone on day 1, and this was compared with two test treatments defined as (1) midazolam with midostaurin on day 3 (to test for inhibition of CYP3A4 by midostaurin) and (2) midazolam alone on day 8 (to test for induction of CYP3A4 by midostaurin). Following log transformation, the primary PK parameters were analyzed using a linear, mixed-effects model using the term of treatment as a fixed factor (reference, test 1, test 2) and subject as a random factor. A point estimate and the corresponding 90 % CI for the difference in test 1 versus reference and the difference in test 2 versus reference were derived from the respective contrasts of the model estimates and anti-logged to obtain the GMR and respective 90 % CI.

### Bioanalytical methods

The concentrations of unchanged midostaurin, CGP52421, and CGP62221 in the plasma were determined using a validated liquid chromatography–tandem mass spectrometry (LC–MS/MS) assay with a lower limit of quantitation (LLOQ) of 10 ng/mL (Novartis SAS France bioanalytics and SGS, Cephac Europe, SAS). The concentration of ketoconazole in plasma was assessed using a validated LC–MS/MS assay with an LLOQ of 50.0 ng/mL (WuXi Pharmatech Co, Ltd, China). The concentration of rifampicin in plasma was measured using a validated LC–MS/MS assay with an LLOQ of 5.0 ng/mL (SGS, Cephac Europe, SAS). Plasma concentrations of midazolam and its metabolite 1′-hydroxymidazolam, which is generated by CYP3A4 and CYP3A5 in humans, were measured using a validated LC–MS/MS assay with an LLOQ of ≈0.100 ng/mL (SGS, Cephac Europe, SAS). The plasma concentration of 4β-hydroxycholesterol was measured using a validated LC–MS/MS assay with an LLOQ of 3.0 ng/mL (SGS, Cephac Europe, SAS). The concentrations of 6β-hydroxycortisol and cortisol in urine were determined using validated LC–MS/MS assays with an LLOQ of 10.0 ng/mL for 6β-hydroxycortisol and 1.0 ng/mL for cortisol. The ratio of 6β-hydroxycortisol to cortisol was subsequently calculated (SGS, Cephac Europe, SAS). See Supplementary Table 2 for detailed bioanalytical methods for all 3 studies.

### Ethics

All studies were conducted in accordance with the Declaration of Helsinki, and all volunteers provided written informed consent according to institutional guidelines. All studies were conducted at the Early Phase Clinical Unit of PAREXEL International GmbH in Berlin, Germany. All study protocols were reviewed and approved by the State Office of Health and Social Affairs Ethics Committee of Berlin (Landesamt für Gesundheitund Soziales Ethik-Kommission des Landes Berlin) for PAREXEL International GmbH (DDI with ketoconazole, EudraCT 2008-003038-39; DDI with rifampicin, EudraCT 2009-009895-11; DDI with midazolam, EudraCT 2009-009870-29).

## Results

### Demographics of healthy volunteers

These 3 studies enrolled 114 healthy volunteers. In all 3 studies, there were 3 populations: the randomized set, the safety set, and the PK set. The randomized set included all participants who were randomized. The safety set included all participants who received at least 1 dose of study drug. The PK set included all participants who received all scheduled doses of study drug and had evaluable PK data; participants who vomited within 4 h after receiving study drug were excluded from the PK set.

The study of midostaurin and ketoconazole enrolled 47 healthy volunteers with a median age of 44 years (range 20–55 years); 62 % of participants were male. Twenty participants were randomized to receive midostaurin alone, and 27 participants were randomized to receive midostaurin plus ketoconazole. All 47 individuals received ≥1 dose of study drug and were included in the safety population. DDIs were assessed in the PK population, which included 36 participants who completed all scheduled doses of study drug and provided evaluable PK profiles (18 participants each in the midostaurin and midostaurin plus ketoconazole arms).

The study of midostaurin and rifampicin enrolled 47 healthy volunteers with a median age of 43 years (range 19–53 years); 60 % of volunteers were male. Twenty-two participants were randomized to receive midostaurin alone, and 25 participants were randomized to receive midostaurin plus rifampicin. The safety population included 46 individuals who received ≥1 dose of study drug. DDIs were assessed in the PK population, which included 40 participants who completed all scheduled doses of study drug and provided evaluable PK profiles (20 participants each in the midostaurin plus rifampicin and midostaurin only arms).

The study of midostaurin and midazolam enrolled 20 healthy volunteers with a median age of 38 years (range 21–54 years); 55 % of volunteers were male. All 20 individuals received ≥1 dose of study drug and were included in the safety population. DDIs were assessed in the PK population, which included 18 participants who completed all scheduled doses of study drug and provided evaluable PK profiles. Baseline characteristics were similar across all arms of the 3 studies (Supplementary Table 3).

### PK results

#### Midostaurin PK when administered with ketoconazole

**Fig. 1:**
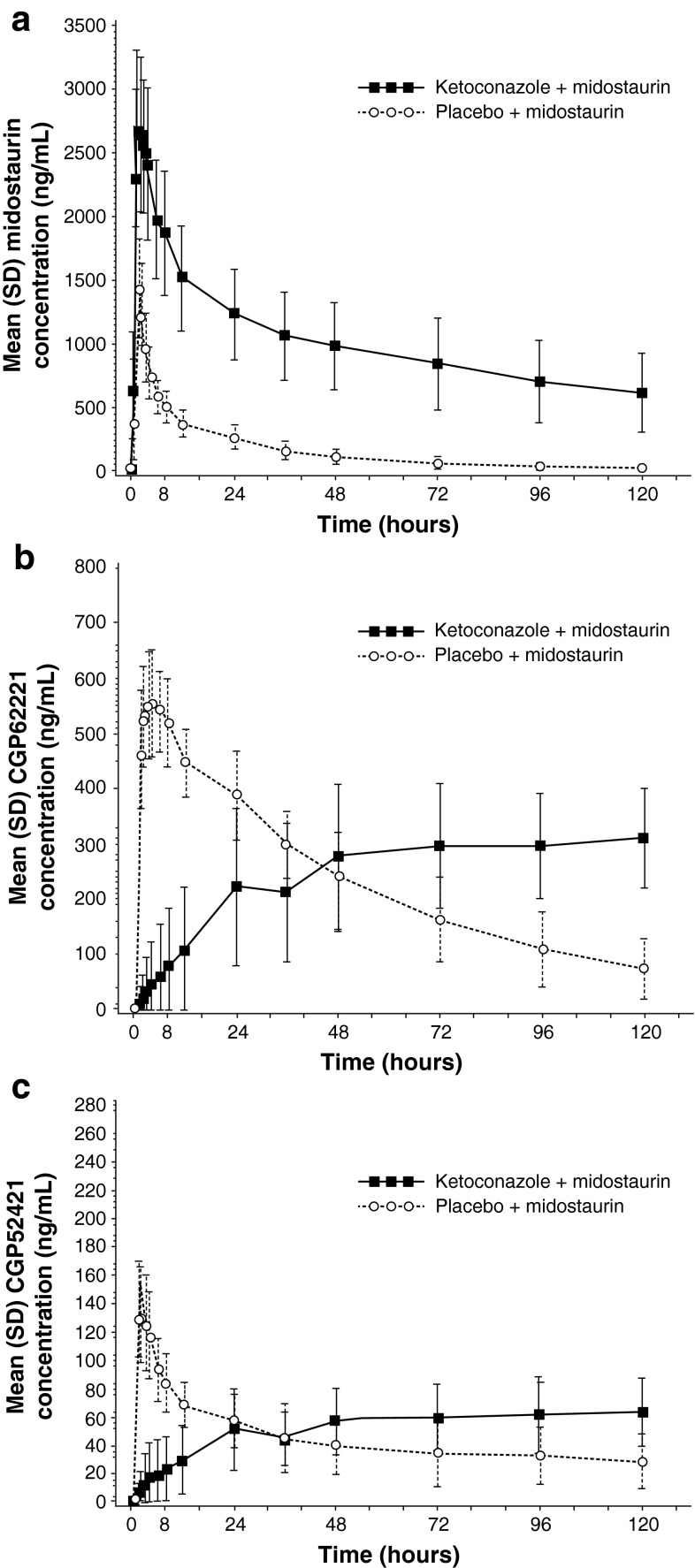
Arithmetic means of the plasma concentration–time profiles of **a** midostaurin, **b** CGP62221, and **c** CGP52421 after oral administration of midostaurin 50 mg daily with placebo or ketoconazole 400 mg daily to healthy volunteers

The effects of the potent CYP3A4 inhibitor ketoconazole on midostaurin PK are shown in Table 2 and Fig. 1. Following inhibition of CYP3A4 by ketoconazole, the *C*_max_ of midostaurin increased by ≈1.8-fold and the AUC increased by tenfold compared with placebo. The *C*_max_ of midostaurin’s metabolites decreased by twofold with ketoconazole compared with placebo. In the presence of ketoconazole, the elimination *t*_1/2_ of midostaurin and CGP62221 were prolonged, indicating that both the formation of this metabolite and its subsequent clearance were inhibited. The fraction of midostaurin metabolized by CYP3A4 (Fm_CYP3A4_) was 91 %, calculated by the following equation:

$${\text{Fm}}_{{{\text{CYP3A}}4}} = 1 - {\text{AUC}}_{{{\text{inf}}}} ({\text{without}}\,{\text{ketoconazole}})/{\text{AUC}}_{{{\text{inf}}}} ({\text{with}}\,{\text{ketoconazole}}) = 1 - (19,762/205,911) = 0.91.$$

**Table 2:** Summary of plasma PK parameters of midostaurin, CGP52421, and CGP62221 in healthy volunteers after administration of midostaurin 50 mg daily with (a) placebo or ketoconazole 400 mg daily or (b) placebo or rifampicin 600 mg daily

PK parameter	Treatment^a^	*n*	Midostaurin		CGP62221		CGP52421	
			Adjusted geometric mean^a^	GMR^a^ (90 % CI)	Adjusted geometric mean^a^	GMR^a^ (90 % CI)	Adjusted geometric mean^a^	GMR^a^ (90 % CI)
(a) Effects of ketoconazole on midostaurin PK (PK population *n* = 36)								
AUC_0–inf_ (ng h/mL)	Ketoconazole + midostaurin	18	205,911.74	10.42 (7.46, 14.56)	110,234.87	3.51 (2.48, 4.98)	NR	NR
	Placebo + midostaurin	18	19,762.50		31,366.63		NR	
AUC_0–last_ (ng h/mL)	Ketoconazole + midostaurin	18	115,423.53	6.10 (4.96,7.51)	27,237.87	0.99 (0.82, 1.21)	5,917.05	1.21 (0.95, 1.54)
	Placebo + midostaurin	18	18,909.36		27,414.44		4,877.88	
*C*_max_ (ng/mL)	Ketoconazole + midostaurin	18	2,892.62	1.83 (1.62, 2.05)	329.76	0.56 (0.48, 0.66)	70.89	0.49 (0.42, 0.58)
	Placebo + midostaurin	18	1,585.02		586.78		144.59	
*t*_max_ (h)	Ketoconazole + midostaurin	18	1.50	0.5 (0, 0.5)	119.67	114.0 (92.8, 116.0)	119.69	116.7 (93.9, 117.9)
	Placebo + midostaurin	18	1.00		3.51		2.00	
*t*_1/2_ (h)	Ketoconazole + midostaurin	18	90.62	NR	142.01^b^	NR	NR	NR
	Placebo + midostaurin	18	23.33		36.60		NR	
(b) Effects of rifampicin on midostaurin PK (PK population *n* = 40)								
AUC_0–inf_ (ng h/mL)	Placebo + midostaurin	20	22,799.27	0.06 (0.05–0.07)	35,146.80	0.08 (0.06–0.09)	NR	NR
	Rifampicin + midostaurin	20	1,347.13		2,699.43		NR	
AUC_0–last_ (ng h/mL)	Placebo + midostaurin	20	22,032.39	0.06 (0.05–0.07)	32,266.80	0.08 (0.06–0.09)	14,634.32	0.41 (0.36–0.46)
	Rifampicin + midostaurin	20	1,230.38		2,501.48		5,962.87	
*C*_max_ (ng/mL)	Placebo + midostaurin	20	1,621.26	0.27 (0.23–0.31)	631.46	0.63 (0.56–0.70)	196.46	0.65 (0.59–0.72)
	Rifampicin + midostaurin	20	440.32		396.25		127.87	
*t*_max_ (h)	Placebo + midostaurin	20	1.02	−0.48 (−0.50 to −0.02)	3.00	−1.50 (−2.00 to −1.50)	3.00	−0.98 (−1.47 to 0.00)
	Rifampicin + midostaurin	20	1.00		1.50		2.51	
*t*_1/2_ (h)	Placebo + midostaurin	20	23.04	NR	33.74	NR	174.95	NR
	Rifampicin + midostaurin	20	5.14		7.02		87.14	

All study drugs were given orally. All doses were given once daily unless otherwise noted. Participants in the PK population received all scheduled doses without vomiting within 4 h after dosing. One patient in the midostaurin 50-mg arm of the rifampicin study did not receive study drug; all other randomized participants not in the PK set did not complete all scheduled dosing without vomiting within 4 h after dosing

*AUC* area under the concentration curve, *GMR* geometric mean ratio, *NR* not reported, *PK* pharmacokinetics, *t*_1/2_ half-life, *t*_max_ time to reach maximal concentration

^a^For *t*_max_, the median is presented under “adjusted geometric mean,” the Hodges–Lehmann estimate of median difference is presented under “GMR,” and 90 % distribution-free CI is presented under “90 % CI”

^b^For 4 volunteers only; other volunteers had a flat terminal phase that made *t*_1/2_ impossible to calculate

### Midostaurin PK when administered with rifampicin

**Fig. 2:**
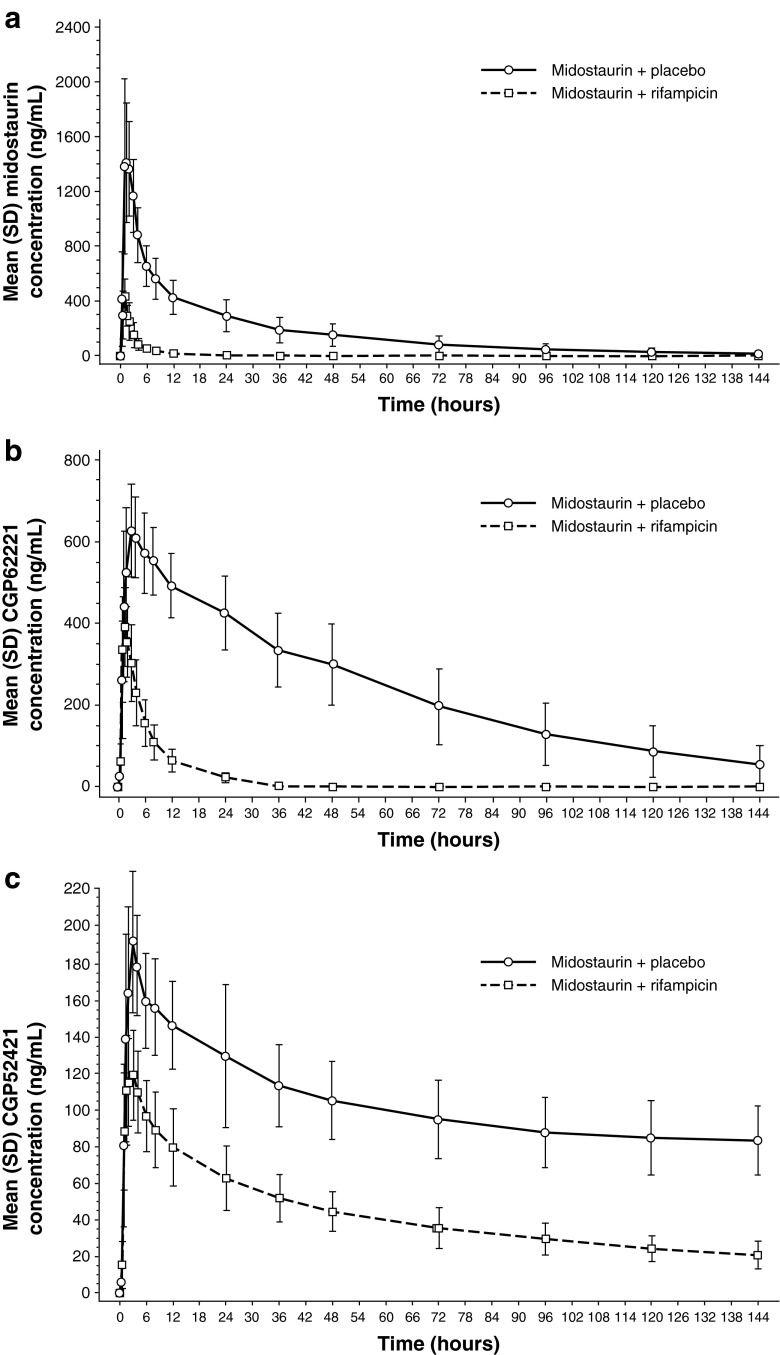
Arithmetic means of the plasma concentration–time profiles of **a** midostaurin, **b** CGP62221, and **c** CGP52421 after oral administration of midostaurin 50 mg daily with placebo or rifampicin 600 mg daily to healthy volunteers

The effects of the CYP3A4 inducer rifampicin on the plasma concentration–time profiles of midostaurin, CGP62221, and CGP52421 are shown in Table 2 and Fig. 2. Co-administration of rifampicin with midostaurin notably decreased *C*_max_ and AUC of midostaurin, with an increase in the geometric mean of the apparent clearance of midostaurin by 16.9-fold on average. Both metabolites, CGP62221 and CGP52421, exhibited the same pattern as midostaurin, showing decreases in *C*_max_ and AUC when midostaurin was co-administered with rifampicin. Median *t*_max_ was prolonged by twofold for CGP62221 and by 1.2-fold for CGP52421 in the midostaurin + placebo arm compared with the midostaurin + rifampicin arm. In the midostaurin + rifampicin arm, the exposure (AUC_last_) for CGP62221 and CGP52421 decreased by 13.0- and 2.45-fold, respectively.

To confirm that the decreased exposure of midostaurin observed was a result of CYP3A4 induction, the levels of 4β-hydroxycholesterol in plasma and the 6β-hydroxycortisol/cortisol ratio in urine were evaluated. Individuals treated with rifampicin are known to have a dose-related increase in the level of 4β-hydroxycholesterol, with low intraindividual variability [21, 22]. In the midostaurin + rifampicin group, geometric mean levels of 4β-hydroxycholesterol increased from day 1 [22.03 ng/mL; coefficient of variance (CV%), 36.45] to day 9 (74.35 ng/mL; CV%, 27.17) and continued to increase to day 15 (102.70 ng/mL; CV%, 25.54). Additionally, there was an ≈4.7-fold increase in the geometric mean 6β-hydroxycortisol/cortisol ratio from day 1 to day 15. In contrast, in the midostaurin + placebo group, no notable change in geometric mean levels or variability of 4β-hydroxycholesterol occurred (day 1 25.33 ng/mL; CV% 34.25; day 9 23.38 ng/mL; CV% 34.60; day 15 23.28 ng/mL; CV% 36.27), and the 6β-hydroxycortisol/cortisol ratio did not increase from day 1 to day 15.

#### Midazolam PK when administered with midostaurin

**Fig. 3:**
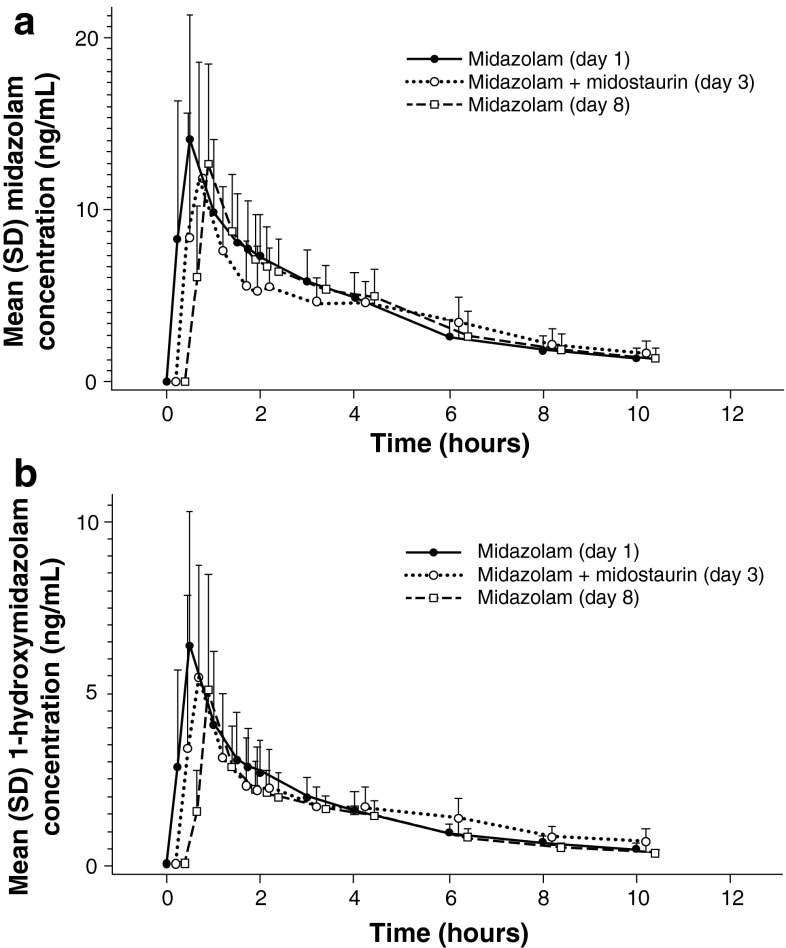
Arithmetic means of the plasma concentration–time profiles of **a** midazolam and **b** 1′-hydroxymidazolam on day 1 (midazolam 4 mg, alone), day 3 (midazolam 4 mg + midostaurin 100 mg), and day 8 (midazolam 4 mg, alone). All study drugs were administered orally. The concentration–time profiles for midazolam + midostaurin (day 3) and midazolam (day 8) have been plotted with a slight shift of time to enable better viewing of the differences between the treatment days

The inhibition assessment on day 3 showed that co-administration of midostaurin with midazolam decreased the *C*_max_ of both midazolam and 1′-hydroxymidazolam by an average of 1.2-fold compared with the *C*_max_ values for midazolam alone, as administered on day 1 (Table 3; Fig. 3). No change in AUC_inf_ was observed for midazolam or 1′-hydroxymidazolam. In the induction assessment (midazolam administered alone on day 8 after repeated doses of midostaurin), average decreases in *C*_max_ of 1.1- and 1.3-fold were observed for midazolam and 1′-hydroxymidazolam, respectively, compared with the *C*_max_ values for midazolam alone, as administered on day 1. Also, average decreases in AUC_inf_ of 1.1- and 1.3-fold were observed for midazolam and 1′-hydroxymidazolam, respectively. Furthermore, repeated dosing of midostaurin was not accompanied by any notable changes in the plasma concentrations of 4β-hydroxycholesterol or in the 6β-hydroxycortisol/cortisol ratio excreted in urine. Therefore, these results suggest that the parent drug midostaurin is neither an inhibitor nor inducer of CYP3A4 in vivo in humans.

**Table 3:** Summary of plasma PK parameters of midazolam and 1′-hydroxymidazolam in healthy volunteers (*n* = 18) after administration of midazolam 4 mg (days 1, 3, and 8) and midostaurin 100 mg (days 3–6)

PK parameter	Treatment^a^	Midazolam		1′-Hydroxymidazolam	
		Adjusted geometric mean^a^	GMR^a^ (90 % CI)	Adjusted geometric mean^a^	GMR^a^ (90 % CI)
AUC_0–inf_ (ng h/mL)	Midazolam (day 1)	49.69		18.18	
	Midazolam + midostaurin (day 3)	49.56	1.00 (0.92–1.08)^b^	18.54	1.02 (0.93–1.12)^b^
	Midazolam (day 8)	46.96	0.95 (0.87–1.02)^c^	13.74	0.76 (0.69–0.83)^c^
AUC_0–last_ (ng h/mL)	Midazolam (day 1)	43.12		15.91	
	Midazolam + midostaurin (day 3)	40.21	0.93 (0.88–0.99)^b^	15.87	1.00 (0.92–1.08)^b^
	Midazolam (day 8)	40.81	0.95 (0.89–1.01)^c^	12.57	0.79 (0.73–0.85)^c^
*C*_max_ (ng/mL)	Midazolam (day 1)	13.50		5.83	
	Midazolam + midostaurin (day 3)	11.02	0.82 (0.67–1.00)^b^	4.76	0.82 (0.63–1.06)^b^
	Midazolam (day 8)	12.25	0.91 (0.74–1.11)^c^	4.37	0.75 (0.58–0.98)^c^
*t*_max_ (h)	Midazolam (day 1)	0.50		0.50	
	Midazolam + midostaurin (day 3)	0.50	−0.02 (−0.74 to 1.00)^b^	0.050	0.13 (−0.50 to 1.50)^b^
	Midazolam (day 8)	0.50	0.12 (−0.12 to 0.26)^c^	0.50	0.01 (−0.23 to 0.26)^c^

All study drugs were given orally. All doses were given once daily unless otherwise noted. Participants in the PK population received all scheduled doses without vomiting within 4 h after dosing. All randomized participants not in the PK set did not complete all scheduled dosing without vomiting within 4 h after dosing

*AUC* area under the concentration curve, *GMR* geometric mean ratio, *NR* not reported, *PK* pharmacokinetics, *t*_max_ time to reach maximal concentration

^a^For *t*_max_, the median is presented under “adjusted geometric mean,” the Hodges–Lehmann estimate of median difference is presented under “GMR,” and 90 % distribution-free CI is presented under “90 % CI”

^b^GMR for day 3/day 1 comparison

^c^GMR for day 8/day 1 comparison

### Safety

In all 3 studies, midostaurin either as single agent or in combination was generally well tolerated, and most AEs resolved rapidly upon discontinuation of study drug. All AEs are presented in Supplementary Table 4.

#### Midostaurin administered with ketoconazole

A total of 34 participants (72 %) reported 15 AEs; nausea was the most frequently reported AE (40 %). Of the 15 AEs reported, 8 AEs (dry skin, flatulence, abdominal distension, cough, nasopharyngitis, pharyngolaryngeal pain, sleep disorder, and somnolence) occurred only in the ketoconazole + midostaurin arm, which had the highest exposure to midostaurin. Six AEs (nausea, diarrhea, dizziness, vomiting, headache, and rhinitis) occurred in both treatment arms, and 1 AE (feeling cold) was reported only in the midostaurin + placebo arm. All AEs were mild in intensity, and all but one (common cold, which was treated with ibuprofen) were resolved without additional therapy. There were no serious AEs, but 7 participants (15 %) discontinued the study because of AEs (6 vomiting and 1 common cold).

#### Midostaurin administered with rifampicin

Twenty-five participants (100 %) in the midostaurin + rifampicin arm and 13 participants (62 %) in the midostaurin arm experienced an AE. In the midostaurin + rifampicin arm, the most frequently reported AEs were chromaturia (96 %; commonly associated with rifampicin therapy), nausea (48 %), diarrhea (20 %), and headache (20 %). In the midostaurin + placebo arm, the most frequently reported AEs were nausea [8 (38 %)] and diarrhea [7 (33 %)]; 1 individual experienced a nervous system AE (headache). In the midostaurin + rifampicin arm, 36 % of participants had nervous system AEs (headache in 5 individuals, dizziness in 4, and somnolence in 2).

#### Midazolam administered with midostaurin

Twelve of 20 enrolled participants (60 %) in the safety set reported AEs. The highest incidence of AEs (45 %) was observed on day 3, when midazolam and midostaurin were co-administered. The most frequently reported AEs were nausea (40 %) and headache (40 %). The majority of the AEs were of CTC grade 1 (*n* = 11) or grade 2 (*n* = 1, nausea) intensity and were transient.

## Discussion

The influence of ketoconazole on midostaurin PK was significant (Fm_CYP3A4_ = 91 %), as demonstrated by the large increase in midostaurin exposure during co-administration with ketoconazole. The relative increase of 1.8-fold in *C*_max_ and tenfold in AUC indicated that most of this effect was due to a decrease in systemic clearance of midostaurin rather than an increase in bioavailability. This DDI can be classified as strong (greater than fivefold increase) based on a proposed interaction classification by the Pharmaceutical Research and Manufacturers of America for drugs biotransformed via CYP3A4 [25].

As anticipated based on the ketoconazole study, CYP3A4 induction with rifampicin had a pronounced impact in the opposite direction on midostaurin exposure. When midostaurin was taken with rifampicin, the geometric mean of the apparent clearance of midostaurin was increased by an average of 16.9-fold compared with midostaurin administered alone. The active metabolites CGP62221 and CGP52421 showed a similar pattern. Co-administration of midostaurin and rifampicin resulted in decreases in the apparent exposures of CGP62221 and CGP52421 by 13.0- and 2.45-fold, respectively, compared with midostaurin alone. These results suggest that midostaurin as well as its metabolites, CGP62221 and CGP52421, are principally cleared from the body through metabolism by CYP3A4. Considering the extent of DDI with a potent CYP3A4 inducer, co-administration of strong or moderate CYP3A4 inducers should be avoided.

Midostaurin, administered as a single dose or in multiple doses, did not appear to affect the concentrations of midazolam or its metabolite 1′-hydroxymidazolam. As a result, it could be concluded that midostaurin is neither a CYP3A4 inhibitor nor a CYP3A4 inducer in humans at current clinically relevant conditions. However, a definitive conclusion cannot be made for the midostaurin metabolites CGP62221 and CGP52421 because of their low exposure following a single dose or 4–5 days of daily midostaurin dosing. Increasing the length of our PK studies was not feasible due to safety concerns in healthy volunteers, but an ongoing phase II study of midostaurin in patients with ASM showed a significant increase in the level of the endogenous CYP3A4 biomarker 4-β-hydroxycholesterol in plasma following 1–2 months of daily midostaurin dosing. These results in patients with ASM suggest CYP3A4 induction, probably by the long half-life metabolite CGP52421 [18].

In these studies, the administration of midostaurin, either alone or in combination, was generally well tolerated by healthy volunteers. All AEs were grade 1 with the exception of two grade 2 events (nausea and common cold).

In summary, these studies showed that midostaurin is a “victim” but not a “perpetrator” of CYP3A4. The inhibition or induction potentials for midostaurin metabolites CGP62221 and CGP52421 could not be fully characterized due to the long half-lives of these two metabolites in humans. The results of these studies provide further guidance on the proper use of midostaurin in clinical settings. The PK profile of midostaurin, characterized in these and other studies, supports its further evaluation in patients with ASM and AML, including the ongoing global phase II study of midostaurin in patients with ASM or mast cell leukemia with or without an associated clonal hematologic non-mast cell lineage disease [16], and the ongoing randomized phase III trial of induction and consolidation chemotherapy with midostaurin or placebo in treatment-naive patients with FLT3-mutated AML [Cancer and Leukemia Group B 10603; Randomized AML Trial in FLT3 in <60-year-olds (RATIFY)] [12].

## Electronic supplementary material

Below is the link to the electronic supplementary material.

Supplementary material 1 (DOC 426 kb)
