# Supplementary material for: Investigation into CYP3A4-mediated drug–drug interactions on midostaurin in healthy volunteers
Source: Cancer Chemother Pharmacol. 2013 Oct 2;72(6):1223–34. doi: 10.1007/s00280-013-2287-6 (PMC3834177; doi:10.1007/s00280-013-2287-6)
Supplement: Supplementary file 1 — Supplementary material 1 (DOC 426 kb) [file 280_2013_2287_MOESM1_ESM.doc]

### Supplementary Table 1. Key enrollment criteria

| **Midostaurin + Ketoconazole Study** | **Midostaurin + Rifampicin Study** | **Midostaurin + Midazolam Study** |
| --- | --- | --- |
| *Inclusion criteria* | | |
| Healthy adults, aged 18-55 years | Healthy adults, aged 18-55 years | Healthy adults, aged 18-55 years |
| Adhering to appropriate contraception | Adhering to appropriate contraception | Adhering to appropriate contraception |
| Body weight 50-90 kg | Body weight 50-90 kg | Body weight 50-90 kg |
| BMI 18-30 kg/m^2^ | BMI 18-29.9 kg/m^2^ | BMI 18-29.9 kg/m^2^ |
| Normal vital signs, including blood pressure | Normal vital signs, including blood pressure | Normal vital signs, including blood pressure |
| Normal laboratory values for liver and renal parameters | Normal laboratory values for liver and renal parameters | Normal laboratory values for liver and renal parameters |
| No other clinical trials within 5 months of study completion | No other clinical trials within 3 months of study completion | No other clinical trials within 5 months of study completion |
| Provided informed consent | Provided informed consent | Provided informed consent |
| Able to comply with dietary, fluid, and lifestyle restrictions | Able to comply with dietary, fluid, and lifestyle restrictions | Able to comply with dietary, fluid, and lifestyle restrictions |
| *Exclusion criteria* | | |
| Breast-feeding or pregnant women | Breast-feeding or pregnant women | Breast-feeding or pregnant women |
| Family history of long QT syndrome | Family history of long QT syndrome | Family history of long QT syndrome |
| Cardiac abnormalities (eg, QT prolongation, atrial or ventricular arrhythmia) | Cardiac abnormalities (eg, QT prolongation, atrial or ventricular arrhythmia) | Cardiac abnormalities (eg, QT prolongation, atrial or ventricular arrhythmia) |
| History of myocardial infarction, angina pectoris, atherosclerosis, or other clinically significant heart disease (eg, congestive heart failure, uncontrolled hypertension) | History of myocardial infarction, angina pectoris, atherosclerosis, or other clinically significant heart disease (eg, congestive heart failure, uncontrolled hypertension) | History of myocardial infarction, angina pectoris, atherosclerosis, or other clinically significant heart disease (eg, congestive heart failure, uncontrolled hypertension) |
| Positive test for HIV, hepatitis B, or hepatitis C | Positive test for HIV, hepatitis B, or hepatitis C | Positive test for HIV, hepatitis B, or hepatitis C |
| Use of prescription medication within 14 days or over-the-counter medication within 7 days | Use of prescription medication within 14 days or over-the-counter medication within 7 days | Use of prescription medication within 14 days or over-the-counter medication within 7 days |

BMI, body mass index.

### Supplementary Table 2. Detailed mass spectrometry methods

|  | | **Midostaurin** |
| --- | --- | --- |
| Internal standard | | ^13^C_6_-midostaurin |
| Range of calibration curve (LLOQ), ng/mL | | 10.0-5000 (10) |
| Performance | Cs  Bias %  CV% | n = 4  −1.4-1.6  1.55-5.83 |
|  |  |  |
|  |  |  |
|  | QCs  Bias %  CV% | n = 4  4.75-10.4  2.11-3.21 |
| Quantification settings (LC conditions) | Column | Luna, PFP(2), 3 μm, 100 Å, 150 × 2 mm |
|  | Column temperature, °C | ≈ +40 |
|  | Autosampler temperature, °C | ≈ +10 |
|  | Injection volume, μL | 10 |
|  | Needle wash | Water/acetonitrile (50:50; vol/vol) with 0.1% formic acid |
|  | Injector wash cycles |  |
|  | Rinsing volume, μL | 450 |
|  | Rinsing speed, μL/s | 35 |
|  | Rinsing dip time, s | 1 |
|  | Rinse mode | Before and after aspiration |
|  | Mobile phase | 5 mM ammonium acetate with 0.1% formic acid/ acetonitrile (40:60; vol/vol) |
|  | Flow rate, μL/min | 300 |
|  | Pump mode | Isocratic mode |
|  | Retention time (approximation), min | Midostaurin and ^13^C_6_-midostaurin: 4.3  CGP62221 and ^13^C_6_-CGP62221: 3.1 |
|  | Total run time, min | 6 |
|  | Pump | LC-20AD pump, Shimadzu |
|  | Degasser | DGU-20A3, Shimadzu |
|  | Autosampler | SIL-20AC autosampler, Shimadzu |
|  | Oven | CTO-10AS oven, Shimadzu |
| Quantification settings (MS conditions) | Mass spectrometer | API3000, Applied Biosystems |
|  | MS/MS interface | TurboIonSpray, Applied Biosystems |
|  | Polarity | Positive |
|  | Resolution Q1 | UNIT |
|  | Resolution Q3 | UNIT |
|  | Midostaurin transition, m/z  Dwell time, ms  ^13^C_6_-midostaurin transition, m/z  Dwell time, ms  CGP62221 transition, m/z  Dwell time, ms  ^13^C_6_-CGP62221 transition, m/z  Dwell time, ms | 571.2-348.0  200  577.4-348.0  200  557.2-348.2  200  563.3-348.2  200 |
|  | TurboIonSpray voltage, V | 2500 |
|  | Interface temperature, °C | 550 |

Cs, calibration standards; CV%, coefficient of covariance; LC, liquid chromatography; LLOQ, lower limit of quantitation; MS, mass spectrometry; QCs, quality controls.

|  | | **CGP62221 (*LC/MS settings same as midostaurin*)** |
| --- | --- | --- |
| Internal standard | | ^13^C_6_-CGP62221 |
| Range of calibration curve (LLOQ), ng/mL | | 10.0-5000 (10) |
| Performance | Cs  Bias %  CV% | n = 4  −2.4-1.0  0.16-4.84 |
|  |  |  |
|  |  |  |
|  | QCs  Bias %  CV% | n = 4  6.75-11.67  1.22-4.32 |
| Quantification settings (LC conditions) | Column | Luna, PFP(2), 3 μm, 100 Å, 150 × 2 mm |
|  | Column temperature, °C | ≈ +40 |
|  | Autosampler temperature, °C | ≈ +10 |
|  | Injection volume, μL | 10 |
|  | Needle wash | Water/acetonitrile (50:50; vol/vol) with 0.1% formic acid |
|  | Injector wash cycles |  |
|  | Rinsing volume, μL | 450 |
|  | Rinsing speed, μL/s | 35 |
|  | Rinsing dip time, s | 1 |
|  | Rinse mode | Before and after aspiration |
|  | Mobile phase | 5 mM ammonium acetate with 0.1% formic acid/ acetonitrile (40:60; vol/vol) |
|  | Flow rate, μL/min | 300 |
|  | Pump mode | Isocratic mode |
|  | Retention time (approximation), min | Midostaurin and ^13^C_6_-midostaurin: 4.3  CGP62221 and ^13^C_6_-CGP62221: 3.1 |
|  | Total run time, min | 6 |
|  | Pump | LC-20AD pump, Shimadzu |
|  | Degasser | DGU-20A3, Shimadzu |
|  | Autosampler | SIL-20AC autosampler, Shimadzu |
|  | Oven | CTO-10AS oven, Shimadzu |
| Quantification settings (MS conditions) | Mass spectrometer | API3000, Applied Biosystems |
|  | MS/MS interface | TurboIonSpray, Applied Biosystems |
|  | Polarity | Positive |
|  | Resolution Q1 | UNIT |
|  | Resolution Q3 | UNIT |
|  | Midostaurin transition, m/z  Dwell time, ms  ^13^C_6_-midostaurin transition, m/z  Dwell time, ms  CGP62221 transition, m/z  Dwell time, ms  ^13^C_6_-CGP62221 transition, m/z  Dwell time, ms | 571.2-348.0  200  577.4-348.0  200  557.2-348.2  200  563.3-348.2  200 |
|  | TurboIonSpray voltage, V | 2500 |
|  | Interface temperature, °C | 550 |

Cs, calibration standards; CV%, coefficient of covariance; LC, liquid chromatography; LLOQ, lower limit of quantitation; MS, mass spectrometry; QCs, quality controls.

|  | | **CGP52421** |
| --- | --- | --- |
| Internal standard | | ^13^C_6_- CGP52421 |
| Range of calibration curve (LLOQ), ng/mL | | 10.0-5000 (10) |
| Performance | Cs  Bias %  CV% | n = 16  −0.8-4.4  2.39-4.15 |
|  |  |  |
|  |  |  |
|  | QCs  Bias %  CV% | n = 16  −1.75-2.40  4.26-5.38 |
| Quantification settings (LC conditions) | Column  Guard column | Alltima C18, 5 µm, 50 × 2.1 mm  Alltima C18, 5 µm, 7.5 × 4.6 mm |
|  | Column temperature, °C | ≈ +60 |
|  | Autosampler temperature, °C | ≈ +10 |
|  | Injection volume, μL | 10 |
|  | Wash 1 | Isopropanol/acetonitrile/methanol/water (25:25:25:25; vol/vol/vol/vol) with 2.5% formic acid |
|  | Wash 2 | Water/acetonitrile (50:50; vol/vol) with 0.1% formic acid |
|  | Injector wash cycles | 2 cleans with wash 1 (100 µL) after injection 1 clean with wash 2 (100 µL) after injection 2 valve cleans with wash 1 (100 µL) after injection 2 valve cleans with wash 2 (100 µL) after injection |
|  | Mobile phase | 5 mM ammonium acetate /acetonitrile (33:67; vol/vol) |
|  | Flow rate, μL/min | 250 |
|  | Pump mode | Isocratic mode |
|  | Retention time (approximation), min | CGP52421 and ^13^C_6_-CGP52421: 1.4 min |
|  | Total run time, min | 6 |
|  | Polarity | Positive |
|  | Resolution Q1 | UNIT |
|  | Resolution Q3 | UNIT |
|  | CGP52421 transition, m/z  Dwell time, ms  ^13^C_6_- CGP52421 transition, m/z  Dwell time, ms | 587.2-364.1  1200  593.2-364.2  200 |
|  | TurboIonSpray voltage, V | 2100 |
|  | Interface temperature, °C | 550 |

Cs, calibration standards; CV%, coefficient of covariance; LC, liquid chromatography; LLOQ, lower limit of quantitation; MS, mass spectrometry; QCs, quality controls.

|  | | **Rifampicin** |
| --- | --- | --- |
| Internal standard | | Rifapentine |
| Range of calibration curve (LLOQ), ng/mL | | 1-250 (1.00) |
| Performance | Cs  Bias %  CV% | n = 4  −3.0-5.0  2.46-6.48 |
|  |  |  |
|  |  |  |
|  | QCs  Bias %  CV% | n = 4  −9.4-3.67  0.93-4.28 |
| Quantification settings  (LC conditions) | Column | Kromasil C18, 5 μm, 150 × 4.6 mm |
|  | Column temperature, °C | Room temperature |
|  | Autosampler temperature, °C | ≈ +10 |
|  | Injection volume, μL | 10 |
|  | Needle wash 1 | Methanol/water (90:10; vol/vol) with 1% formic acid |
|  | Needle wash 2 | Acetonitrile/isopropanol/water (60:20:20; vol/vol/vol) with 1% formic acid |
|  | Injector wash cycles | 1 flush of wash 1 and 1 flush of wash 2 (100 μL) before injection  2 flushes of wash 1 and 2 flushes of wash 2 (100 μL) after injection |
|  | Valve clean | 1 flush of wash 1 and 1 flush of wash 2 (100 μL) |
|  | Mobile phase | Acetonitrile/10 mM ammonium formate with 0.05% formic acid (45:55; vol/vol) |
|  | Flow rate, μL/min | 1000 |
|  | Pump mode | Isocratic mode |
|  | Split to MS interface, μL/min | 400 |
|  | Retention time (approximation), min | Rifampicin: 3  Rifapentine: 6 |
|  | Total run time, min | 9 |
|  | Pump | 1100 binary pump, Agilent |
|  | Degasser | Series 1100 degasser, Agilent |
|  | Autosampler | HTS PAL autosampler, CTC Analytics |
| Quantification settings  (MS conditions) | Mass spectrometer | API 3000, Applied Biosystems |
|  | MS/MS interface | TurboIonSpray, Applied Biosystems |
|  | Polarity | Positive |
|  | Resolution Q1 | UNIT |
|  | Resolution Q3 | UNIT |
|  | Rifampicin transition, m/z  Dwell time, ms  Rifapentine transition, m/z  Dwell time, ms | 823.5-791.4  1000  877.5-845.4  1000 |
|  | TurboIonSpray voltage, V | 3500 |
|  | Interface temperature, °C | 550 |

Cs, calibration standards; CV%, coefficient of covariance; LC, liquid chromatography; LLOQ, lower limit of quantitation; MS, mass spectrometry; QCs, quality controls.

|  | | **Ketoconazole** |
| --- | --- | --- |
| Internal standard | | Ketoconazole-D8 |
| Range of calibration curve (LLOQ), ng/mL | | 50-15 000 (50) |
| Performance | Cs  Bias %  CV% | n = 12  −6.0-4.7  2.0-5.6 |
|  |  |  |
|  |  |  |
|  | QCs  Bias %  CV% | n = 28  −2.5-6.7  2.8-3.9 |
| Quantification settings (LC conditions) | Column | CAPCELL PAK MG C18, 5 μm (2.0 mm I.D. × 50 mm), Shiseido |
|  | Column temperature, °C | +40 |
|  | Autosampler temperature, °C | +8 |
|  | Injection volume, μL | 20 |
|  | Injector wash cycles |  |
|  | Wash A | 0.2% TFA in acetonitrile |
|  | Wash B | Methanol |
|  | Mobile phase | Solution A: 0.2% TFA in water  Solution B: ACN |
|  | Syringe wash | 2 × injector wash A, 2 × injector wash B |
|  | Valve wash | 5 × injector wash A, 5 × injector wash B |
|  | Flow rate, μL/min | 400 |
|  | Pump mode | Isocratic over 3.5 min with mobile phase B set at 30% |
|  | Retention time (approximation), min | Ketoconazole 1.50  Ketoconazole-D8: 1.45 |
|  | Curtain gas | 20 |
|  | GS1 | 40 |
|  | GS2 | 55 |
|  | Interface heater | ON |
|  | Collision gas collision activation | 6 unit |
|  | Mode | Electrospray ionization, Multiple reaction monitoring, Positive ion |
|  | Ketoconazole transition, m/z  Ketoconazole-D8 transition, m/z | 531.2-489.1  539.2-497.1 |
|  | SourceIonSpray voltage, V | 1500 |
|  | Interface temperature, °C | 550 |

ACN, acetonitrile/water; Cs, calibration standards; CV%, coefficient of covariance; LC, liquid chromatography; LLOQ, lower limit of quantitation; MS, mass spectrometry; QCs, quality controls; TFA, trifluoroacetic acid.

|  | | **Midazolam** | **1-Hydroxymidazolam** |
| --- | --- | --- | --- |
| Internal standard | | Alprazolam | |
| Range of calibration curve (LLOQ), ng/mL | | 0.100-100 (0.100) | |
| Performance | Cs  Bias %  CV% | n = 20-22  −1.8-2.0  3.34-5.74 | n = 21-22  −3.6-4.0  2.81-5.89 |
|  |  |  |  |
|  |  |  |  |
|  | QCs  Bias %  CV% | n = 22  −2.0-0.13  4.35-6.82 | n = 22  0-3.38  4.89-6.57 |
| Quantification settings (LC conditions) | Column | Chromolith performance RP-18e, 100 × 4.6 mm | |
|  | Column temperature, °C | ≈ + 40 | |
|  | Autosampler temperature, °C | Room temperature | |
|  | Injection volume, μL | 30 | |
|  | Needle wash | Isopropanol/Milli-Q water (70:30; v/v) with 1% formic acid | |
|  | Injector wash cycles |  | |
|  | Flush volume | 250 μL, 1 flush before injection and 3 flushes after injection | |
|  | Mobile phase | Ammonium acetate 5 mM/acetonitrile (60:40; v/v) with 0.1% of acetic acid | |
|  | Flow rate, μL/min | 500 | |
|  | Pump mode | Isocratic | |
|  | Retention time (approximation), min | Midazolam: 7.4  1-Hydroxymidazolam: 6.1  Alprazolam: 6.3 | |
|  | Total run time, min | 14 | |
|  | Pump | Series 1100 binary pump, Agilent | |
|  | Degasser | Series 1100 degasser, Agilent | |
|  | Autosampler | Series 200 autosampler, Perkin Elmer | |
|  | Oven | Croco-Cil, CIL | |
| Quantification settings (MS conditions) | Mass spectrometer | API3000, Applied Biosystems | |
|  | MS/MS interface | APCI (heated nebulizer), Applied Biosystems | |
|  | Polarity | Positive | |
|  | Resolution Q1 | UNIT | |
|  | Resolution Q3 | UNIT | |
|  | Midazolam transition, m/z  Dwell time, ms  1-hydroxymidazolam transition, m/z  Dwell time, ms  Alprazolam transition, m/z  Dwell time, ms | 326.2-291.2  700  342.2-324.2  700  309.3-281.1  200 | |
|  | Interface temperature, °C | 550 | |

Cs, calibration standards; CV%, coefficient of covariance; LC, liquid chromatography; LLOQ, lower limit of quantitation; MS, mass spectrometry; QCs, quality controls.

|  | | | **4β-Hydroxycholesterol** | |
| --- | --- | --- | --- | --- |
| Internal standard | | | 4β-Hydroxycholesterol-D7 | |
| Range of calibration curve (LLOQ), ng/mL | | | 3.00-250 (3.00) | |
| Performance | Cs  Bias %  CV% | | n = 6  −3.5-7.33  1.75-8.95 | |
|  |  |  |  |  |
|  |  |  |  |  |
|  | QCs  Bias %  CV% | | n = 6  −5.47-0.8  3.94-7.43 | |
| Quantification settings  (LC conditions) | Column  In-line column filter | | Kinetex, C18, 100 Å, 2.6 μm, 2.1 × 150 mm  HPLC KrudKatcher ultra in-line filter 0.5 μm | |
|  | Column temperature, °C | | ≈ +55 | |
|  | Autosampler temperature, °C | | ≈ +10 | |
|  | Injection volume, μL | | 10 | |
|  | Needle wash | | Isopropanol/methanol (80:20; vol/vol) + 1% formic acid | |
|  | Injector wash cycles | | 3 flushes (100 μL) to wash the syringe and  3 flushes (100 μL) to wash the injection port after injection | |
|  | Mobile phase A | | 5 mM ammonium acetate in methanol/water (80:20; vol/vol) | |
|  | Mobile phase B | | 5 mM ammonium acetate in methanol | |
|  | Flow rate, μL/min | | 300 | |
|  | Pump mode | | Gradient   \| Total Time, min \| Mobile Phase A, % \| Mobile Phase B, % \| \| --- \| --- \| --- \| \| 0.0 \| 20 \| 80 \| \| 2.0 \| 20 \| 80 \| \| 2.1 \| 0 \| 100 \| \| 3.5 \| 0 \| 100 \| \| 3.6 \| 20 \| 80 \| \| 10.0 \| 20 \| 80 \| | |
|  | Auxiliary mobile phase | | Methanol | |
|  | Auxiliary flow rate, μL/min | | 300 | |
|  | Switching valve | | Column to mass spectrometer for 1-4 min | |
|  | Retention time (approximation), min | | 4β-Hydroxycholesterol: 2.7  4β-Hydroxycholesterol-D7: 2.7 | |
|  | Total run time, min | | 10 | |
|  | Pump  Auxiliary pump | | Series 1100 binary pump (without mixing column)  Series 200 pump, Perkin Elmer | |
|  | Degasser | | Series 1100 degasser, Agilent | |
|  | Autosampler | | HTC PAL autosampler, CTC Analytics | |
|  | Oven | | Croco-Cil, Cluzeau Info Labo; External switch valve, Rheodyne | |
| Quantification settings  (MS conditions) | Mass spectrometer | | Quattro Ultima Platinum, Waters (Micromass) | |
|  | MS/MS interface | | Z-Spray, Waters (Micromass) | |
|  | Polarity | | Positive | |
|  | Resolution MS1 (LM and HM) | | 14.5 | |
|  | Resolution MS2 (LM and HM) | | 14.5 | |
|  | 4β-Hydroxycholesterol transition, m/z  Dwell time, ms  Collision energy, eV  4β-Hydroxycholesterol-D7 transition, m/z  Dwell time, ms  Collision energy, eV | | 402.50-385.50  250  10  409.60-392.50  250  10 | |
|  | ZSpray voltage, V | | 5000 | |
|  | Source temperature (desolvation temperature), °C | | 150 (300) | |
|  | | | **6β-Hydroxycortisol** |  |
| Internal standard | | | Hydroxycortisol-D3 |  |
| Range of calibration curve (LLOQ), ng/mL | | | 10-3000 (10) |  |
| Performance | | Cs  Bias %  CV% | n = 6  −8.0-5.8  1.71-4.85 |  |
|  |  |  |  |  |
|  |  |  |  |  |
|  |  | QCs  Bias %  CV% | n = 6  −5.33-3.6  1.66-3.17 |  |
| Quantification settings  (LC conditions) | | Column  Guard column | Chromolith RP-18e, 100 × 4.6 mm, Merck  Chromolith RP-18e, 5 × 4.6 mm, Merck |  |
|  |  | Column temperature, °C | +30 |  |
|  |  | Autosampler temperature, °C | +10 |  |
|  |  | Injection volume, μL | 20 |  |
|  |  | Needle wash | Methanol/water (50:50; vol/vol) |  |
|  |  | Injector wash cycles |  |  |
|  |  | Rinsing volume, μL | 450 |  |
|  |  | Rinsing speed, μL/s | 35 |  |
|  |  | Rinsing dip time, s | 1 |  |
|  |  | Rinse mode | Before and after aspiration |  |
|  |  | Volume of flushes, μL | 100 |  |
|  |  | Flushes before injection | 2 |  |
|  |  | Flushes after injection | 4 |  |
|  |  | Valve clean | 2 |  |
|  |  | Mobile phase A | Water/acetonitrile (90:10; vol/vol) with 0.5% formic acid |  |
|  |  | Mobile phase B | Water/acetonitrile (80:20; vol/vol) with 0.5% formic acid |  |
|  |  | Split to MS interface, μL/min | 500 |  |
|  |  | Pump mode | Gradient   \| Time, min \| Flow rate, µL/min \| Mobile Phase A, % \| Mobile Phase B, % \| \| --- \| --- \| --- \| --- \| \| 0.00 \| 2000 \| 100 \| 0 \| \| 4.00 \| 2000 \| 100 \| 0 \| \| 4.01 \| 2000 \| 0 \| 100 \| \| 11.50 \| 2000 \| 0 \| 100 \| \| 11.51 \| 3000 \| 0 \| 100 \| \| 13.50 \| 3000 \| 0 \| 100 \| \| 13.51 \| 3000 \| 100 \| 0 \| \| 15.50 \| 3000 \| 100 \| 0 \| \| 15.51 \| 2000 \| 100 \| 0 \| \| 16.00 \| 2000 \| 100 \| 0 \| |  |
|  |  | Auxiliary mobile phase | Water/acetonitrile (90/10; vol/vol) with 0.5% formic acid |  |
|  |  | Auxiliary pump flow rate, μL/min | 2000 |  |
|  |  | Retention time (approximation), min | 6β-Hydroxycortisol: 3  6β-Hydroxycortisol-D3: 3 |  |
|  |  | Total run time, min | 16 |  |
|  |  | Pump  Auxiliary pump | Series LC-20AD, Shimadzu and Series 1100 binary pump, Agilent  Series 200 pump, Perkin Elmer |  |
|  |  | Degasser | DGU-20A3, Shimadzu and Series 1100, Agilent |  |
|  |  | Autosampler | SIL-20AC, Shimadzu and HTS PAL autosampler, CTC Analytics |  |
|  |  | Oven | CTO-10AS, Shimadzu and TCM/CHM, Waters; External switch valve, Valco |  |
| Quantification settings  (MS conditions) | | Mass spectrometer | API4000, Applied Biosystems |  |
|  |  | MS/MS interface | TurboIonSpray, Applied Biosystems |  |
|  |  | Polarity | Positive |  |
|  |  | Resolution Q1 | UNIT |  |
|  |  | Resolution Q3 | UNIT |  |
|  |  | 6β-Hydroxycortisol transition, m/z  Dwell time, ms  Collision energy, eV  6β-Hydroxycortisol-D3 transition, m/z  Dwell time, ms  Collision energy, eV | 379.3-343.2  500  19  382.2-346.3  100  19 |  |
|  |  | TurboIonSpray voltage, V | 5000 |  |
|  |  | Interface temperature, °C | 750 |  |

Cs, calibration standards; CV%, coefficient of covariance; LC, liquid chromatography; LLOQ, lower limit of quantitation; MS, mass spectrometry; QCs, quality controls.

|  | | **Cortisol (*Same as 6β-hydroxycortisol*)** |
| --- | --- | --- |
| Internal standard | | Cortisol-D4 |
| Range of calibration curve (LLOQ), ng/mL | | 1.00-100 (100) |
| Performance | Cs  Bias %  CV% | n = 6  −2.40-2.50  1.86-6.73 |
|  |  |  |
|  |  |  |
|  | QCs  Bias %  CV% | n = 6  2.13-4.40  1.87-4.64 |
| Quantification settings  (LC conditions) | Column  Guard column | Chromolith RP-18e, 100 × 4.6 mm, Merck  Chromolith RP-18e, 5 × 4.6 mm, Merck |
|  | Column temperature, °C | +30 |
|  | Autosampler temperature, °C | +10 |
|  | Injection volume, μL | 20 |
|  | Needle wash | Methanol/water (50:50; vol/vol) |
|  | Injector wash cycles |  |
|  | Rinsing volume, μL | 450 |
|  | Rinsing speed, μL/s | 35 |
|  | Rinsing dip time, s | 1 |
|  | Rinse mode | Before and after aspiration |
|  | Volume of flushes, μL | 100 |
|  | Flushes before injection | 2 |
|  | Flushes after injection | 4 |
|  | Valve clean | 2 |
|  | Mobile phase A | Water/acetonitrile (90:10; vol/vol) with 0.5% formic acid |
|  | Mobile phase B | Water/acetonitrile (80:20; vol/vol) with 0.5% formic acid |
|  | Split to MS interface, μL/min | 500 |
|  | Pump mode | Gradient   \| Time, min \| Flow rate, µL/min \| Mobile Phase A, % \| Mobile Phase B, % \| \| --- \| --- \| --- \| --- \| \| 0.00 \| 2000 \| 100 \| 0 \| \| 4.00 \| 2000 \| 100 \| 0 \| \| 4.01 \| 2000 \| 0 \| 100 \| \| 11.50 \| 2000 \| 0 \| 100 \| \| 11.51 \| 3000 \| 0 \| 100 \| \| 13.50 \| 3000 \| 0 \| 100 \| \| 13.51 \| 3000 \| 100 \| 0 \| \| 15.50 \| 3000 \| 100 \| 0 \| \| 15.51 \| 2000 \| 100 \| 0 \| \| 16.00 \| 2000 \| 100 \| 0 \| |
|  | Auxiliary mobile phase | Water/acetonitrile (90/10; vol/vol) with 0.5% formic acid |
|  | Auxiliary pump flow rate, μL/min | 2000 |
|  | Retention time (approximation), min | Cortisol: 9.8  Cortisol-D4: 9.8 |
|  | Total run time, min | 16 |
|  | Pump  Auxiliary pump | Series LC-20AD, Shimadzu and Series 1100 binary pump, Agilent  Series 200 pump, Perkin Elmer |
|  | Degasser | DGU-20A3, Shimadzu and Series 1100, Agilent |
|  | Autosampler | SIL-20AC, Shimadzu and HTS PAL autosampler, CTC Analytics |
|  | Oven | CTO-10AS, Shimadzu and TCM/CHM, Waters; External switch valve, Valco |
| Quantification settings  (MS conditions) | Mass spectrometer | API4000, Applied Biosystems |
|  | MS/MS interface | TurboIonSpray, Applied Biosystems |
|  | Polarity | Positive |
|  | Resolution Q1 | UNIT |
|  | Resolution Q3 | UNIT |
|  | Cortisol transition, m/z  Dwell time, ms  Collision energy, eV  Cortisol-D4 transition, m/z  Dwell time, ms  Collision energy, eV | 363.3-121.0  500  36  367.3-121.0  100  36 |
|  | TurboIonSpray voltage, V | 5000 |
|  | Interface temperature, °C | 750 |

Cs, calibration standards; CV%, coefficient of covariance; LC, liquid chromatography; LLOQ, lower limit of quantitation; MS, mass spectrometry; QCs, quality controls.

|  | | **4β-Hydroxycholesterol** |
| --- | --- | --- |
| Internal standard | | 4β-Hydroxycholesterol-D7 |
| Range of calibration curve (LLOQ), ng/mL | | 3.00-250 (3.00) |
| Performance | Cs  Bias %  CV% | n = 6  −3.5-7.33  1.75-8.95 |
|  |  |  |
|  |  |  |
|  | QCs  Bias %  CV% | n = 6  −5.47-0.8  3.94-7.43 |
| Quantification settings  (LC conditions) | Column  In-line column filter | Kinetex, C18, 100 Å, 2.6 μm, 2.1 × 150 mm  HPLC KrudKatcher ultra in-line filter 0.5 μm |
|  | Column temperature, °C | ≈ +55 |
|  | Autosampler temperature, °C | ≈ +10 |
|  | Injection volume, μL | 10 |
|  | Needle wash | Isopropanol/methanol (80:20; vol/vol) + 1% formic acid |
|  | Injector wash cycles | 3 flushes (100 μL) to wash the syringe and  3 flushes (100 μL) to wash the injection port after injection |
|  | Mobile phase A | 5 mM ammonium acetate in methanol/water (80:20; vol/vol) |
|  | Mobile phase B | 5 mM ammonium acetate in methanol |
|  | Flow rate, μL/min | 300 |
|  | Pump mode | Gradient   \| Total Time, min \| Mobile Phase A, % \| Mobile Phase B, % \| \| --- \| --- \| --- \| \| 0.0 \| 20 \| 80 \| \| 2.0 \| 20 \| 80 \| \| 2.1 \| 0 \| 100 \| \| 3.5 \| 0 \| 100 \| \| 3.6 \| 20 \| 80 \| \| 10.0 \| 20 \| 80 \| |
|  | Auxiliary mobile phase | Methanol |
|  | Auxiliary flow rate, μL/min | 300 |
|  | Switching valve | Column to mass spectrometer for 1-4 min |
|  | Retention time (approximation), min | 4β-Hydroxycholesterol: 2.7  4β-Hydroxycholesterol-D7: 2.7 |
|  | Total run time, min | 10 |
|  | Pump  Auxiliary pump | Series 1100 binary pump (without mixing column)  Series 200 pump, Perkin Elmer |
|  | Degasser | Series 1100 degasser, Agilent |
|  | Autosampler | HTC PAL autosampler, CTC Analytics |
|  | Oven | Croco-Cil, Cluzeau Info Labo; External switch valve, Rheodyne |
| Quantification settings  (MS conditions) | Mass spectrometer | Quattro Ultima Platinum, Waters (Micromass) |
|  | MS/MS interface | Z-Spray, Waters (Micromass) |
|  | Polarity | Positive |
|  | Resolution MS1 and MS2 (LM and HM) | 14.5 |
|  | 4β-Hydroxycholesterol transition, m/z  Dwell time, ms  Collision energy, eV  4β-Hydroxycholesterol-D7 transition, m/z  Dwell time, ms  Collision energy, eV | 402.50-385.50  250  10  409.60-392.50  250  10 |
|  | ZSpray voltage, V | 5000 |
|  | Source temperature (desolvation temperature), °C | 150 (300) |

Cs, calibration standards; CV%, coefficient of covariance; LC, liquid chromatography; LLOQ, lower limit of quantitation; MS, mass spectrometry; QCs, quality controls.

### Supplementary Table 3. Baseline patient demographics

|  | **Midostaurin + Ketoconazole Study** | | | **Midostaurin + Rifampicin Study** | | | **Midostaurin + Midazolam Study** |
| --- | --- | --- | --- | --- | --- | --- | --- |
|  | **Midostaurin**  **50 mg**  **(n = 20)** | **Midostaurin**  **50 mg +**  **Ketoconazole**  **400 mg**  **(n = 27)** | **All Participants**  **(N = 47)** | **Midostaurin**  **50 mg +**  **Rifampicin**  **600 mg**  **(n = 25)** | **Midostaurin 50 mg**  **(n = 22)** | **All Participants**  **(N = 47)** | **All Participants**  **Midostaurin 100 mg once daily + Midazolam 4 mg**  **N = 20** |
| Median age (range), y | 46 (20-55) | 43 (21-55) | 44 (20-55) | 36 (19-52) | 47 (30-53) | 43 (19-53) | 38 (21-54) |
| Male, n (%) | 11 (55) | 18 (67) | 29 (62) | 15 (60) | 13 (59) | 28 (60) | 11 (55) |
| White, n (%) | 20 (100) | 26 (96.3) | 46 (97.9) | 24 (95) | 21 (96) | 45 (96) | 20 (100) |
| Median weight,  kg (range) | 78.0  (63.5-89.9) | 72.4  (57.8-88.5) | 74.0  (57.8-89.9) | 76.2  (55.0-88.8) | 78.3  (57.3-88.8) | 77.3  (55.0-88.8) | 69.7  (51.2-88.9) |
| Median height, cm (range) | 175  (165-198) | 171  (165-186) | 174  (165-198) | 176  (159-187) | 175  (156-189) | 176  (156-189) | 173  (158-188) |
| Median BMI, kg/m^2^ (range) | 24.8  (21.0-29.1) | 23.6  (18.0-29.2) | 24.0  (18.0-29.2) | 24.3  (21.0-29.6) | 25.1  (19.7-29.9) | 24.7  (19.7-29.9) | 23.4  (19.6-27.0) |
| Randomized, n | 20 | 27 | 47 | 25 | 22 | 47 | 20 |
| Safety population,^a^ n | 20 | 27 | 47 | 25 | 21 | 46 | 20 |
| PK population,^b^ n | 18 | 18 | 36 | 20 | 20 | 40 | 18 |

All study drugs were given orally. All doses were once daily unless otherwise noted. Patients in the PK population received all scheduled doses without vomiting within 4 hours after dosing. One patient in the midostaurin 50-mg arm of the rifampicin study did not receive study drug; all other randomized patients not in the PK set did not complete all scheduled dosing without vomiting within 4 hours after dosing.

BMI, body mass index; PK, pharmacokinetics.

^a^ Received ≥ 1 dose of study drug.

^b^ Completed all scheduled doses of study drug and provided evaluable PK profiles.

### Supplementary Table 4. Adverse events regardless of study drug relationship

| **Preferred Term** | **Mido**  **50 mg**  **(n = 20)** | **Mido**  **50 mg +**  **Keto**  **400 mg**  **(n = 27)** | **All Participants**  **(N = 47)** | **Mido**  **50 mg +**  **Rifa**  **600 mg**  **(n = 25)** | **Mido 50 mg**  **(n = 21)** | **All Participants**  **(N = 46)** | **MZ 4 mg** | **MZ 4 mg + Mido 100 mg** | **Mido**  **50 mg twice daily** | **MZ 4 mg** | **All Participants (N = 20)** |
| --- | --- | --- | --- | --- | --- | --- | --- | --- | --- | --- | --- |
|  | **n (%)** | **n (%)** | **n (%)** | **n (%)** | **n (%)** | **n (%)** | **n (%)** | **n (%)** | **n (%)** | **n (%)** | **n (%)** |
| Any preferred term | 13 (65.0) | 21 (77.8) | 34 (72.3) | 25 (100.0) | 13 (61.9) | 38 (82.6) | 3 (15.0) | 9 (45.0) | 7 (38.9) | 3 (16.7) | 12 (60.0) |
| Nausea | 7 (35.0) | 12 (44.4) | 19 (40.4) | 12 (48.0) | 8 (38.1) | 20 (43.5) | 0 | 7 (35.0) | 2 (11.1) | 0 | 8 (40.0) |
| Diarrhea | 4 (20.0) | 10 (37.0) | 14 (29.8) | 5 (20.0) | 7 (33.3) | 12 (26.1) | 0 | 2 (10.0) | 1 (5.6) | 0 | 3 (15.0) |
| Dizziness | 5 (25.0) | 4 (14.8) | 9 (19.1) | 4 (16.0) | 0 | 4 (8.7) | 0 | 0 | 0 | 0 | 0 |
| Vomiting | 1 (5.0) | 5 (18.5) | 6 (12.8) | 4 (16.0) | 1 (4.8) | 5 (10.9) | 0 | 2 (10.0) | 0 | 0 | 2 (10.0) |
| Dry skin | 0 | 3 (11.1) | 3 (6.4) | 0 | 0 | 0 | 0 | 0 | 0 | 0 | 0 |
| Headache | 2 (10.0) | 1 (3.7) | 3 (6.4) | 5 (20.0) | 1 (4.8) | 6 (13.0) | 2 (10.0) | 2 (10.0) | 5 (27.8) | 2 (11.1) | 8 (40.0) |
| Feeling cold | 2 (10.0) | 0 | 2 (4.3) | 0 | 0 | 0 | 0 | 0 | 0 | 0 | 0 |
| Flatulence | 0 | 2 (7.4) | 2 (4.3) | 1 (4.0) | 1 (4.8) | 2 (4.3) | 0 | 0 | 1 (5.6) | 0 | 1 (5.0) |
| Rhinitis | 1 (5.0) | 1 (3.7) | 2 (4.3) | 1 (4.0) | 0 | 1 (2.2) | 0 | 0 | 0 | 0 | 0 |
| Abdominal distension | 0 | 1 (3.7) | 1 (2.1) | 0 | 0 | 0 | 0 | 0 | 0 | 0 | 0 |
| Cough | 0 | 1 (3.7) | 1 (2.1) | 0 | 0 | 0 | 0 | 0 | 0 | 0 | 0 |
| Nasopharyngitis | 0 | 1 (3.7) | 1 (2.1) | 0 | 2 (9.5) | 2 (4.3) | 0 | 1 (5.0) | 0 | 1 (5.6) | 2 (10.0) |
| Pharyngolaryngeal pain | 0 | 1 (3.7) | 1 (2.1) | 0 | 0 | 0 | 0 | 0 | 0 | 0 | 0 |
| Sleep disorder | 0 | 1 (3.7) | 1 (2.1) | 0 | 0 | 0 | 0 | 0 | 0 | 0 | 0 |
| Somnolence | 0 | 1 (3.7) | 1 (2.1) | 2 (8.0) | 0 | 2 (4.3) | 1 (5.0) | 0 | 0 | 0 | 1 (5.0) |
| Palpitations | 0 | 0 | 0 | 0 | 0 | 0 | 0 | 0 | 1 (5.6) | 0 | 1 (5.0) |
| Feeling hot | 0 | 0 | 0 | 0 | 0 | 0 | 0 | 0 | 1 (5.6) | 0 | 1 (5.0) |
| Restlessness | 0 | 0 | 0 | 0 | 0 | 0 | 0 | 0 | 1 (5.6) | 0 | 1 (5.0) |
| Dyspepsia | 0 | 0 | 0 | 2 (8.0) | 0 | 2 (4.3) | 0 | 0 | 0 | 0 | 0 |
| Discolored feces | 0 | 0 | 0 | 1 (4.0) | 0 | 1 (2.2) | 0 | 0 | 0 | 0 | 0 |
| Back pain | 0 | 0 | 0 | 0 | 1 (4.8) | 1 (2.2) | 0 | 0 | 0 | 0 | 0 |
| Chromaturia | 0 | 0 | 0 | 24 (96.0) | 0 | 24 (52.2) | 0 | 0 | 0 | 0 | 0 |
| Hot flush | 0 | 0 | 0 | 2 (8.0) | 0 | 2 (4.3) | 0 | 0 | 0 | 0 | 0 |

All study drugs were given orally. All doses were once daily unless otherwise noted.

Keto, ketoconazole; mido, midostaurin; MZ, midazolam; rifa, rifampicin.
